# Governance and Public Health Decision-Making During the COVID-19 Pandemic: A Scoping Review

**DOI:** 10.3389/phrs.2024.1606095

**Published:** 2024-02-16

**Authors:** Sumegha Asthana, Sanjana Mukherjee, Alexandra L. Phelan, Claire J. Standley

**Affiliations:** ^1^ Center for Global Health Science and Security, Georgetown University, Washington, DC, United States; ^2^ Heidelberg Institute of Global Health, University of Heidelberg, Heidelberg, Germany

**Keywords:** governance, decision-making, COVID-19, pandemic preparedness, public health governane

## Abstract

**Objective:** We provide an in-depth understanding of how governance and decision-making during the COVID-19 pandemic has been empirically characterized in the literature to identify gaps in research and highlight areas that require further inquiry.

**Methods:** We searched peer-reviewed publications using empirical data published between Jan 1, 2020 and Jan 31, 2022 in three electronic databases to examine the process of governance and decision-making during the COVID-19 pandemic. Two authors independently screened the records and 24 publications were extracted for the review.

**Results:** Governance is analyzed by its level at national, sub-national, community and by its aspects of process, determinants and performance. While different methodological approaches are used, governance is conceptualized in four ways 1) characteristics and elements, 2) leadership, 3) application of power and 4) models or arrangements of governance.

**Conclusion:** For future pandemic preparedness, there is a need for more empirical research using a unified conceptual approach to governance, which integrates decision-making processes and can guide governance structures and mechanisms across different countries and contexts. We call for more inclusivity in who performs the research on governance and where.

## Introduction

Governance is defined as the process of decision-making and implementing (or not implementing) decisions [1]. Governance is relevant to pandemic preparedness at the procedural and substantive levels. Effective governance and public health decision-making, which include key elements of good governance such as accountability, transparency, equity, participation, and rule of law, is necessary for a cohesive pandemic response [2]. Previous studies on governance and public health decision-making during the H1N1 influenza pandemic note the need for transparency and accountability in decision-making [3] and raise concerns about the inadequate integration of scientific advice in the decision-making process [4]. Throughout the course of the COVID-19 pandemic, national governance has emerged as a crucial but often neglected element of preparedness and response [5]. While experts have acknowledged the critical role of governance in health emergency preparedness and response [6], previous methods of assessing health emergency preparedness during the COVID-19 pandemic have primarily focused on country technical capacities (e.g., health system capacity, disease surveillance capacity, etc.) to respond to public health threats [7]. A recent bibliometric analysis of 1,437 articles summarizing the research trends of public health governance of the COVID-19 pandemic highlights three major streams including features of the pandemic and its effects, public health governance regulatory interventions, and evaluation of the effects of the policies [8]. While these streams provide useful insights for improving the overall public health governance of pandemics, comprehensive frameworks for understanding and evaluating the process of pandemic governance and public health decision-making at different levels are limited.

It is important to understand how the pandemic was managed at different levels, including the aspects, elements and functions of its management, and the role of different interest holders in management. Such understanding helps to draw an accurate and complete picture of how governance and decision-making occur during a pandemic to inform recommendations for future preparedness. This scoping review aims to provide an in-depth understanding of how governance and decision-making during the COVID-19 pandemic has been previously characterized in the literature, at the national, sub-national and local levels, to identify gaps in the current research and highlight areas that require further inquiry.

Our main objective is to understand the methodologies and conceptualizations of governance in the existing literature. We do not aim to evaluate the quality of studies or the governance performance of specific countries or draw lessons learned for governance and decision-making during the pandemic. Our research questions are as follows: 1) In which countries has governance and decision-making during the COVID-19 pandemic been studied, 2) How and through what methodological approaches have previous studies analyzed governance and decision-making during the COVID-19 pandemic, and 3) How have previous studies conceptualized governance and decision-making during the COVID-19 pandemic.

## Methods

This scoping review uses Arksey and O’Malley’s (2005) scoping methodology [9]. This framework appropriately captures broad topics that may require a wide range of study designs. While many publications focus on governance and decision-making during the COVID-19 pandemic, we focus on a specific subset of peer-reviewed empirical studies that clearly describe the methodological approaches to assess the process of governance and decision-making during the COVID-19 pandemic. We define empirical data studies as studies in which scholars provide sufficient information to allow the reproduction of their findings (e.g., sampling strategy, data collection, and analysis [10]). We use the terms “governance” and “decision-making” interchangeably in this review.

### Information Sources

Between Feb 1, 2022, and Feb 8, 2022 we searched three electronic databases PubMed, Global Health and EBSCO host Academic Search Premier for peer-reviewed publications, articles, and reports. SA and SM identified relevant databases and developed, pilot-tested and revised the keywords and search strategy in these three databases.

### Search Strategy

The search strategy involved formulating keywords and Medical Subject Headings (MeSH) relevant to our research questions related to “governance,” “policy-making,” “health security,” “pandemic preparedness,” “public health decision-making,” “COVID” (Supplementary Table S1). We restricted searches to peer-reviewed publications in English published between Jan 1, 2020, and Jan 31, 2022. We imported 2,763 references from our database search results to Covidence [11].

### Eligibility Criteria of Included Studies

We included English-language, peer-reviewed, empirical research publications reporting from quantitative, qualitative, or mixed methods studies including descriptive studies, case studies, case series, and research articles. We assessed the relevance of the retrieved studies to ensure that their outcomes relate to the description or analysis of governance and decision-making processes in the COVID-19 pandemic or health emergency at the national, subnational, and local levels focusing on overarching pandemic governance, decisions to break the transmission of the virus, decisions related to diagnosis and treatment, and decisions related to people’s adherence to public health strategies. Because our focus was on studying the process of governance and decision-making, we excluded clinical guideline documents, clinical decision-making studies, and studies focused on policy implementation (Supplementary Table S2).

### Study Selection, Categorization, and Data Extraction

We employed an iterative approach to select, categorize, and extract data from the retrieved publications. Data extraction was conducted by two authors between April and May 2022. After the removal of duplicates from the references, SA and SM independently screened titles and abstracts of the references and included records if they met the criteria stated in Supplementary Table S2. The full text of the included publications was then screened for eligibility. An inductive, analytic approach was used to identify the investigated domains by deriving themes from the publications and related research questions. SA and SM developed a data extraction framework (Supplementary Table S3) in Airtable [12] utilizing 12 columns extraction framework. They extracted data for the first ten rubric examining the region and country studied; income status of the countries as per the low-income countries (LICs); lower-middle-income countries (LMICs); upper-middle-income countries (UMICs); and high-income countries (or HICs) [13]; country and organizational affiliation of authors; type of publication; methodological approach and methods of data collection; and level of governance examined.

For the next two rubrics examining the aspects and conceptualizations of governance, SA reviewed the extracted data and developed a typology by coding data to create themes (Table 1). The typology was developed iteratively as we sought to identify, categorize, and characterize the aspects and conceptualizations of governance. For aspects of governance, three broad themes emerged: 1) process, 2) determinants, and 3) performance. When examining the conceptualization of governance, four broad themes emerged: 1) governance by its characteristics and elements, 2) governance as leadership, 3) governance as the application of power, and 4) governance as models or arrangements of governance. This typology was shared with the entire authorship team and finalized with their feedback. Findings were then clustered under the emerging typology and drafted in the narrative formats.

**TABLE 1:** Typology of aspects and conceptualization of governance (governance and decision-making scoping review, global, 2020–2022).

Typology	Description
Aspects of Governance	
Process	Process implies the mechanism of decision-making and governance to understand “how” governance was planned and delivered
Determinants	Determinant implies the impact of contextual factors or attributes of (effective) decision making and governance to understand “why” governance was (in)effective
Performance	Performance implies the efficiency of governance and characteristics of (effective) governance
Conceptualization of Governance	
Characteristics and elements	Conceptualization of governance by its characteristics and elements implies describing governance through its nature like flexible, inclusive, adaptive and resilient or components like cognition, communication and collaboration
Leadership	Conceptualization of governance as leadership implies describing governance as individual characteristics and personality traits of the political leaders and elected representatives
Application of power	Conceptualization of governance as application of power implies describing governance as exerting or sharing of power by government and other decision-making bodies with other entities and communities
Models or arrangements of governance	Conceptualization of governance as models or arrangements of governance implies administrative structures and units; levels and arrangement of division of responsibilities among the administrative units

## Results

Here we present our findings as per the data extraction framework (Supplementary Table S3) and discuss the emerging typologies of aspects and conceptualization of governance.

**FIGURE 1:**
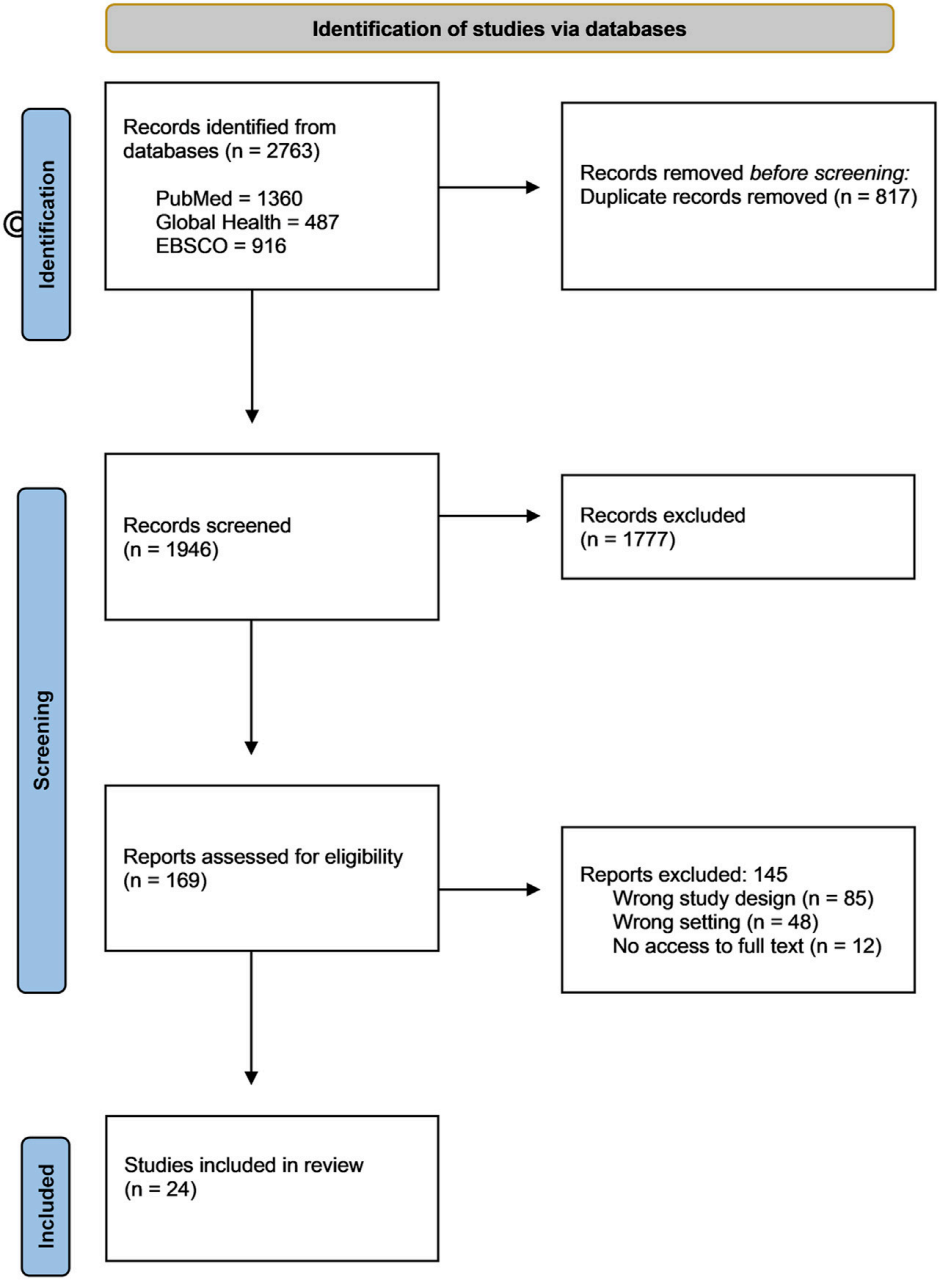
PRISMA flowchart for scoping review (governance and decision-making scoping review, global, 2020–2022).

The database search returned 2,763 records. Of these, 817 duplicates were discarded. We screened the titles and abstracts of the remaining 1,946 records out of which 1,777 records with irrelevant titles and abstracts were excluded, and 145 records were further excluded as they did not meet the inclusion criteria. On full-text review, 24 publications met the inclusion criteria and were included in the study for analysis. The bulk of the excluded articles were either non-empirical articles or were focused on the implementation of policies, public health decisions or clinical decisions during the pandemic. The updated Preferred Reporting Items for Systematic Reviews and Meta Analyses (PRISMA) flow diagram [14] in Figure 1 lays out these procedures in more detail.

### Countries and Regions in Which COVID-19 Pandemic Governance or Decision-Making Has Been Empirically Studied

**FIGURE 2:**
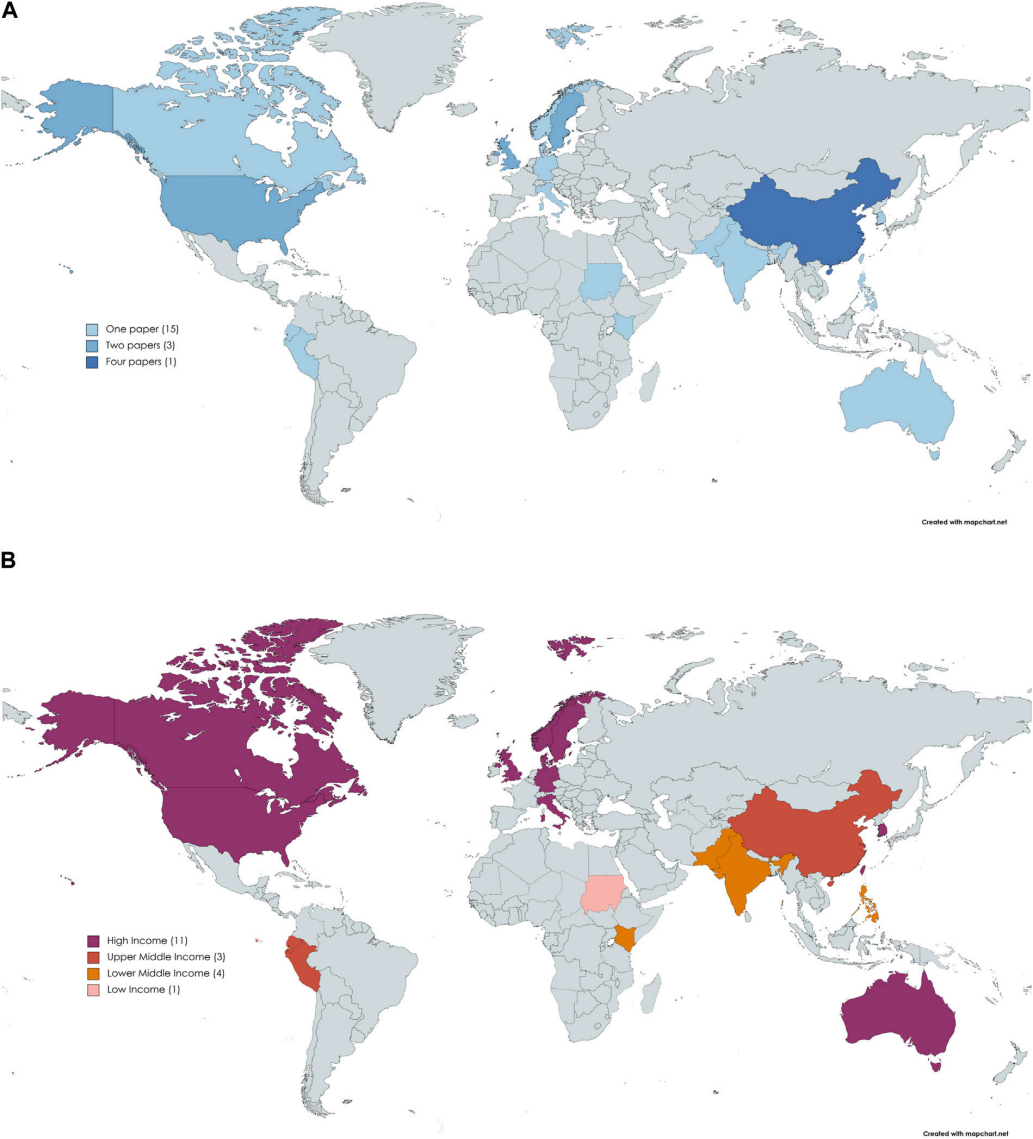
World maps depicting country level analysis. **(A)** World map showing number of papers identified per country studied. **(A)** World map showing countries studied as per income status. All figures were generated using Mapchart.net (https://www.mapchart.net/world.html) (governance and decision-making scoping review, global, 2020–2022). **(A)** World map showing number of papers identified per country studied (governance and decision-making scoping review, global, 2020–2022). **(B)** World map showing countries studied as per income status (governance and decision-making scoping review, global, 2020–2022).

Excluding the 172 countries studied in the six large multi-country publications, 15 countries are included in at least one of the 18 remaining publications. Of these 15 countries, three countries (United Kingdom, United States of America, and Sweden) are included in two publications while four publications focus on China (Figure 2A). In terms of regions, the highest number of publications (*n* = 6) focus on Europe and the Central Asian region, followed by East Asia and the Pacific (*n* = 5). If examined by the income status classification of the countries (Figure 2B), 61% (*n* = 11) of all publications study high-income countries while only one focuses on a low-income country.

### Author Affiliations

For all the 24 publications included in our review, authors were affiliated with the academic sector, mostly universities. Only six out of 24 publications record authors from non-academic backgrounds. Few publications record authors affiliated with the government (*n* = 2) and Civil Society Organizations (*n* = 2). In the two publications, the authors are not affiliated with any organisation. In terms of country affiliations, excluding the six large multi-country publications, most publications record author affiliations with local in-country institutions. However, three publications studying governance and decision-making in LMIC countries do not report affiliation with the local in-country institutions (Table 2).

**TABLE 2:** Author affiliations for publications extracted in our review studying Low- or Middle-Income Country (LMIC) countries (governance and decision-making scoping review, global, 2020–2022).

Country of focus in publication	Country of author affiliation
India	Norway, Sweden
Pakistan	China
Kenya, Sudan, Philippines	Denmark, United Kingdom

### Analysis of Governance and Decision-Making During the COVID-19 Pandemic

Publications have analyzed governance in two main ways. First is by the level of governance, namely, national, regional, sub-national, and community. Second is by the aspect of governance, namely, studying the process, determinants, and performance of governance. Below we describe the levels and aspects that emerged from the findings.

#### Levels of Governance

Included studies focus on one or more levels of governance. A total of 17 publications focus on studying national level governance. Out of these, eight publications examined only one country, whereas three publications are three-country comparison studies. All six of the large multi-country studies included in the review focus on examining governance at the national level. The scope of analysis of the six publications studying large multi-country national-level studies, ranges from examining 26 to 172 countries. Three publications looked at the sub-national level, followed by two at the community level. One publication examines all four levels of governance that is national, regional, sub-national, and community.

#### Aspects of Governance

Our findings indicate that the included studies focus on three main aspects of governance: process of governance, determinants of governance, and performance of governance (Table 1).

##### Process of Governance

Publications focusing on the process of governance examine the mechanism of decision-making and governance to understand “how” governance was planned and delivered. Around 60% of publications in the review (*n* = 14) examine the process of governance and decision-making. These include eight studies focusing on the national level, two examining sub-national issues, two looking at the community level, and one examining all levels of governance.

They focus on processes ranging from studying government actions, administrative conflicts, the role of institutions, the functioning of institutions, and the involvement of stakeholders in decision-making and governance. For example, one publication examines the role of institutions and contexts in shaping crisis management outcomes at the national, regional, sub-national, and local levels [15]. Sub-national level publications focus on studying the mechanism of insufficient resilience in governance [16] and the role of sub-national and national governments in providing protective public health responses [17]. Whereas, the community level publications examine the mechanism of successful collective action [18] and operationalization of local level institutions [19]. National level publications (*n* = 8) study the working of the government ranging from examining the whole model of crisis management [20], to specific aspects like administrative conflicts among federal and provincial governments [21]; governmental consideration of “community participation” [22], the process of community-led responses [23] and deployment of “calculative technologies” by governments [24]. Three out of the 14 publications focusing on the process of governance examine the participation of different stakeholders in policy and governance. The focus of these publications ranges from studying the participation of all actors at different levels [25] to specific stakeholders like health policy and politics researchers [26] and veterinarians [27].

##### Determinants of Governance

Publications focusing on the determinants of governance examine the impact of contextual factors or attributes of (effective) governance and focus on understanding “why” governance was effective or ineffective. About 30% (*n* = 8) of publications examine the determinants of governance. These include six publications focusing on the national level and two on the sub-national level. The national level publications include four large multi-country studies examining the impact of factors like health infrastructure, past experience with pandemics, and governance structure [28]; governance structures and the role of science [29]; attributes of effective disaster response [30]; and differences in the policy preferences of national expert groups [31]. Publications under this category also examine the impact of political contexts like types of regimes [32] and leaders’ personality traits [33]. Sub-national publications examine the impact of leadership roles of mayors [34] and neighborhood social capital and collaborative neighborhood governance [35].

##### Performance of Governance

Publications focusing on the performance of governance examine the efficiency of governance and characteristics of (effective) governance. Our study revealed that 8% (*n* = 2) of all publications focus on the performance of governance. These include two large multi-country studies, one examining the efficiency in the management of health resources [36] and the other examining the characteristics of high and low-performing countries such as coordinating, and strengthening a suite of public health, health system, and socioeconomic measures to prevent or break chains of transmission in communities [37].

### Methodological Approaches to Study the Process of Governance and Decision-Making During the COVID-19 Pandemic

**FIGURE 3:**
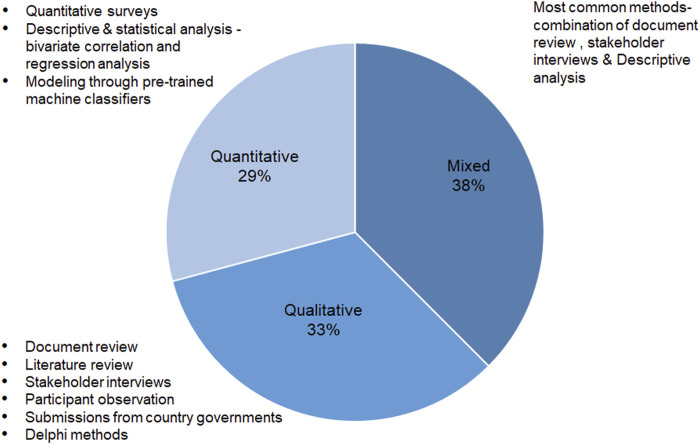
Methodological approach and methods for data collection used in publications (*n* = 24) to study governance and decision-making (governance and decision-making scoping review, global, 2020–2022).

Different methodological approaches and methods for collecting data are used in the included publications (Figure 3). The most commonly used are mixed methods followed by qualitative methods. Nine publications use mixed methods for data collection. The most commonly used method of collecting data in mixed methods studies is a combination of document review and stakeholder interviews. However, methods such as large quantitative surveys are also used in combination with stakeholder interviews and literature reviews for developing country case studies and doing comparative analysis in countries. Eight publications use purely qualitative methods for data collection using a combination of one or more techniques, out of which the most commonly used method is key stakeholder interviews, followed by document analysis. Seven publications are purely quantitative studies using the methods of quantitative surveys, and descriptive and statistical analysis like bivariate correlation and regression analysis. These studies also include modelling through pre-trained machine classifiers.

### Conceptualization of Governance and Decision-Making During the COVID-19 Pandemic

More than 60% (*n* = 16) of all publications mentioned a clear definition or some form of conceptualization of governance. Four broad typologies of governance emerged: 1) conceptualization of governance by its characteristics and elements, 2) conceptualization of governance as leadership, 3) conceptualization of governance as application of power, and 4) conceptualization of governance as models or arrangements of governance (Table 1). These conceptualizations vary from characterizing governance as “adaptive” or “resilient;” “good” governance; characteristics of leadership, or breaking it into levels of multi-level or local or community and neighborhood governance. Others have conceptualized it in terms of the application of power by the government, its administrative structures, and the sharing of power between these administrative structures. Eight publications did not state a clear conceptualization of governance.

#### Conceptualizing Governance by Its Characteristics and Elements

Publications conceptualizing governance by its characteristics mainly characterize governance as “adaptive” and “resilient.” Adaptive governance is defined as “flexible and learning-based multi-level modes of governance or institutional arrangements that can build resilience for the challenges posed by complex and urgent problems” [20]. While adaptive conceptualization is used in one publication to study the whole model of governance at the national level, another publication conceptualizes adaptive governance as “resilience” at the community level in China [16]. They explore the “manifestations … of insufficient resilience in community public health crisis governance, based on the complex adaptive system theory, which emphasizes interaction among subjects and between subjects and the environment to improve the adaptability to the environment” (16 p.1). Another publication examining governance structures, and the role of science and the media during the COVID-19 pandemic in Germany, Sweden, and the United Kingdom uses Blanchet et al’s resilience framework [38] to understand the COVID-19 response. They map “legitimacy, interdependence, knowledge generation, and the capacity to deal with uncertainty” to study preparedness [29].

Other publications conceptualize governance by its quality. For example, a large multi-country study examining the role of governance in purchasing and administering the COVID-19 vaccine in 172 countries, conceptualizes governance as good governance and analyzes the “quality of governance through Governance-related indicators in six areas: voice and accountability, political stability and absence of violence, government effectiveness, regulatory quality, rule of law, and control of corruption” [39]. Others conceptualize governance in terms of its elements. For example, a publication analyses the elements of Governance: cognition, communication, collaboration, and control and suggests confidence (trust in government’s competency) and coproduction (public participation in disaster transmission prevention) for effective disaster response [30].

#### Conceptualizing Governance as Leadership

Other publications conceptualizing governance as “Leadership and Personality,” refer to “individual characteristics that predispose people to act in particular ways, but which also interact with environmental factors (e.g., actions of others, political context) to shape the behavior and decisions of members of political elites” [33]. This study analyses personality traits of openness to experience, conscientiousness, extraversion, agreeableness, and neuroticism or emotional stability, to study stricter and more timely responses to the pandemic [33]. Another study emphasizes the leadership conceptualization of governance as, “Leadership role of the mayor in enacting leadership actions aimed at implementing government ordinances, at coordinating the key actors of local governance, and at (co)producing regular and new public services” [34].

#### Conceptualizing Governance as Application of Power

Some publications conceptualize governance in the view of power and application of power by the government and decision-making bodies. For example, a publication analyzing the United Kingdom government’s population governance strategies uses Foucault’s ideas of “governmentality” and “Bio-politics” to analyze the government’s COVID-19 pandemic strategies. The authors describe governmentality as “the ways in which the state exercises control over or governs, the body of its population” and “bio-politics” as the way for neo-liberal governments to manage their populations and administer the mechanics of life” [24]. Another publication that examines the Ecuadorian government’s legitimization of the exclusion of “community participation” as a value and tenet of health promotion, conceptualizes governance as the application of power by the government in decision-making taking into consideration; or omitting, negating, or distorting “community participation” [22].

Another publication that examines governance in view of power in a rural community in China, conceptualizes it as “Collaborative Leadership and Community Governance” and states “collaborative leadership emphasizes “shared power” or power sharing, rather than authority control through partnership and mutual learning” [18]. Another publication examining collaborative governance in six Chinese cities, emphasizes the exercise of power by the government in the form of hierarchical steering by the government through setting policy priorities and providing support [35].

#### Conceptualizing Governance as Models or Arrangements of Governance

A small proportion of publications (*n* = 2) conceptualize governance in view of the models or arrangements of governance. These include studying the administrative structures, multiple levels of governance arrangements, and sharing of power among different levels of governance. For example, a publication conceptualizes governance in terms of levels, applying Piattoni’s Multi-level governance (MLG) framework [40] to investigate the leadership of China over intergovernmental and private actions in tackling COVID-19. It examines the involvement of different levels and sectors in tackling the COVID-19 pandemic and the roles played by various actors [25]. Another publication conceptualizing governance in terms of levels in Peru applies a “multi-scalar perspective to analyze crisis response within and interactions between” four levels of government [15].

Our results also found a study on the organizational conflicts in Pakistan focusing on public policy implementations conceptualizing governance in terms of the (conflicts between) administrative levels of federal and provincial government [21]. Additionally, another publication focused on centralized and decentralized governance and highlights the structures in view of authority and responsibility “… decentralization, wherein the central government transfers authority and responsibility for specific tasks to lower levels of government” and “Centralized governance helps provide a unified response to a pandemic as a hierarchy economizes on the cost of knowledge production … ” [28]. Others conceptualize governance at the local level and emphasize “by local governance we mean the steering and coordination of relevant actors to achieve public value goals for a given locality” [34]. This study also emphasizes the leadership conceptualization of governance. Another publication focusing on local governance conceptualizes it as the operationalization of local level institutions for disease control and social welfare mechanisms in rural India [19].

## Discussion

We explore how governance and public health decision-making during the COVID-19 pandemic have been studied in the literature. Our study is not intended to evaluate the performance of the countries in governing the pandemic but aims to understand the methods and conceptions used to study the process of governance and public health decision-making. To fulfill this aim, we reviewed the literature to explore where previous research on governance has been undertaken, who is studying governance and decision-making, and how governance and decision-making have been analyzed and conceptualized in the existing literature. Below, we discuss our findings in the context of future preparedness of research, and contributions to literature.

Our review shows that empirical studies present diversity in field in the field of governance as well as disparate methodological choices, ranging from mixed methods to purely qualitative and quantitative approaches. This heterogeneity in research design though reflects the comprehensive understanding of the research topics, it also poses potential challenges in terms of synthesizing findings, ensuring methodological rigour and the generalizability of research findings. Though a move towards mixed research methods leveraging a combination of statistical analysis with document reviews and stakeholder interviews shows advancement in research design, the reliance on large quantitative surveys in conjunction with stakeholder interviews and literature reviews in certain publications, particularly in the context of developing country case studies and comparative analyses, introduces an inherent methodological complexity.

We add to the existing literature on the meaning and process of governance in the global public health setting. Our typology of conceptualization of governance as characteristics, arrangement or structures, application of power and leadership, validates the framework by Berman and colleagues which unites the scholarship around institutional, political, organizational, and governance (IPOG) aspects of the COVID-19 response [41]. Our findings about elements, determinants and performance as the aspects of governance studied during the COVID-19 pandemic complement the three ontological variations noted by Lee and Scott [42] in global health governance scholarship including the scope of institutional arrangements, strengths and weaknesses of existing institutions, and the ideal form and function of global health governance. Our analysis also supports the five strategic approaches to smart governance for health by Kickbusch and Gleicher [43] and confirms their claims that governance is “increasingly conducted across levels, from local to global; regional and local actors therefore have increasing relevance, making effective multilevel governance as important as cross-sectoral and participatory governance” (46 p.10).

Our review points to three main gaps and opportunities in future research on governance and decision-making during the pandemic. Firstly, there is scope for more empirical research examining the process of governance. Out of 169 publications selected for full-text review, 145 were excluded as they did not use empirical research design or did not focus on the “process” of governance. Secondly, there is an opportunity for the practitioners of governance to engage in research on governance. All publications under our review listed authors affiliated with the academic sector. Only two authors were from the government and two were from civil society organizations. Thirdly, there is an opportunity for scholars and practitioners from LMICs to engage and claim space in the research on governance during health emergencies. For all LMIC publications under our review, the authors were based outside the country under study and did not provide details of local affiliations or local partners if any.

Conceptually, three main areas of research could be improved. One, there is a need for more research examining governance as a whole including all levels of governance and interactions among these levels. There is also scope for generating more evidence from the lower levels of governance especially at the community level for future pandemic preparedness. Only one publication in our review examined all levels of governance and only two publications focused on the community level.

Second, the aspects of decision-making focusing on the process of arriving at specific public health decisions taken during the pandemic demands more empirical research. Though there is growing interest among researchers to study broader policy making process including composition of taskforces and its impact on public health decisions [44, 45]; role of contextual factors like political environment, political contestation and population adherence to response measures in decision-making [46]; and significance of translation of policy to practice for decision-making [47]. Robust research providing evidence on the process of arriving at specific decisions, especially during health emergencies, such as prioritization of distribution of resources, decisions related to implementing non-pharmaceutical interventions will provide useful insights on the process of decision-making and opportunities for improving these processes.

Third, and most importantly, there is a need to develop a common and unified understanding and framework for studying governance during pandemics and other health emergencies. Publications included in our review focused on different aspects, parts and levels of governance. There are also variations as per different waves of the pandemic. While these variations provide useful contextual insights into different aspects of governance, a unified approach to study governance will prove useful in comparing governance structures and mechanisms across different countries and contexts. Having unified frameworks for analyzing governance during health emergencies will also be useful for creating generalizable results and guidance for improving governance by lesson-drawing from other contexts.

### Limitations

Although our scoping review is one of the first to provide a comprehensive review of how governance and decision-making during a health emergency such as the COVID-19 pandemic has been studied in literature, it has some limitations. Firstly, our inclusion criteria were limited to peer-reviewed empirical research records in the English language. This criterion limited the inclusion of opinion and perspective articles and also other gray literature in forms of reports and workshops proceedings. Though including opinions, perspectives and reports could add value to this analysis, empirical studies with a clearly defined methodological approach providing sufficient information to allow the reproduction of the findings is important to create evidence on methodological insights and shortcomings in the existing knowledge in this domain. Secondly, the time frame for our review was between Jan 1, 2020, and Jan 31, 2022, limiting the inclusion of important publications after this period, which certainly would add substantial data to this analysis given the increased volume of publications pertaining to COVID-19 during this time period [48]. Thirdly, we recognize the value of examining the results of the studies that were reviewed and propose that we draw policy insights from the included literature. Such analysis necessitates a systematic review of the literature and warrants an investigation into the quality of the publications. We suggest that future systematic reviews should focus on studying the best practices and lessons learned to enhance governance and decision-making during pandemic.

### Conclusion

Our review presents a comprehensive appraisal of the empirical evidence in the area of governance and decision-making during COVID-19 pandemic. Theoretically, our analysis adds to the existing knowledge around understanding the functioning of different levels, components and determinants of governance affecting its performance. Methodologically, our findings provide insights and lessons for designing governance and public health decision-making studies. Pragmatically, our analysis highlights the scope for future empirical research on governance for future pandemic preparedness. It suggests the need for empirical research in the areas of use of evidence in governance and decision-making; methods of effective engagement of different stakeholders; methods of priority setting during health emergencies; and balancing the urgency in the situations of scarcity of scientific evidence. Our analysis calls for a unified conceptual approach to governance, that integrates decision-making processes and more inclusivity in who performs the research on governance and where.
