# Supplementary material for: Governance and Public Health Decision-Making During the COVID-19 Pandemic: A Scoping Review
Source: Public Health Rev. 2024 Feb 16;45:1606095. doi: 10.3389/phrs.2024.1606095 (PMC10904583; doi:10.3389/phrs.2024.1606095)
Supplement: Supplementary file 1 [file DataSheet1.docx]

**Supplementary Material**

The search strategy was designed to retrieve all eligible articles published between Jan 1, 2020, and Jan 31, 2022. The electronic databases PubMed, Global Health and EBSCO host Academic Search Premier were searched using appropriate search terms.

The following search terms were searched: “governance”, “policy-making”, “health security”, “pandemic preparedness”, “pandemic response”, “public health policy”, “health policy”, “public policy”, “social policy”, “global health policy”, “public health decision-making”, “regulations”, “risk management”, “risk prevention”, “policy response”, “pandemic control”, “pandemic management”, “disaster management”, “disaster governance”, “epidemic response”, “epidemic management”, “epidemic governance”, “emergency response”, “emergency management”, “public health measures”, “public health interventions”, “COVID”.

The following Filters and Boolean Search was applied:

**Title filter** (to ensure search words only occurred in the title): “governance” OR “policy-making” OR “health security” OR “pandemic preparedness” OR “pandemic response” OR “public health policy” OR “health policy” OR “public policy” OR “social policy” OR “global health policy” OR “public health decision-making” OR “regulations” OR “risk management” OR “risk prevention” OR “policy response” OR “pandemic control” OR “pandemic management” OR “disaster management” OR “disaster governance” OR “epidemic response” OR “epidemic management” OR “epidemic governance” OR “emergency response” OR “emergency management” OR “public health measures” OR “public health interventions”

**All fields (PubMed)/Keywords (Global Health)/All text (EBSCO Host) filter** (to ensure that the term appeared in all fields and may include MeSH terms): COVID (added with an AND)

**Supplementary Table 1. Digital Search Strategy (governance and decision-making scoping review, global, 2020-2022).**

|  | |
| --- | --- |
| Database/Search Engine | Search Strategy |
| PubMed | [governance] OR [policy-making] OR [health security] OR [pandemic preparedness] OR [pandemic response] OR [public health policy] OR [health policy] OR [public policy] OR [social policy] OR [global health policy] OR [public health decision-making] OR [regulations] OR [risk management] OR [risk prevention] OR [policy response] OR [pandemic control] OR [pandemic management] OR [disaster management] OR [disaster governance] OR [epidemic response] OR [epidemic management] OR [epidemic governance] OR [emergency response] OR [emergency management] OR [public health measures] OR [public health interventions] AND [COVID] |
| Global Health | (governance or (policy adj making) or (health adj security) or (pandemic adj preparedness) or (pandemic adj response) or (public adj health adj policy) or (health adj policy) or (public adj policy) or (social adj policy) or (global adj health adj policy) or (public adj health adj decision adj making) or regulations or (risk adj management) or (risk adj prevention) or (policy adj response) or (pandemic adj control) or (pandemic adj management) or (disaster adj management) or (disaster adj governance) or (epidemic adj response) or (epidemic adj management) or (epidemic adj governance) or (emergency adj response) or (emergency adj management) or (public adj health adj measures) or (public adj health adj interventions)).m_titl. AND COVID.mp. |
| EBSCO host Academic | TI ( governance OR policy-making OR health security OR pandemic preparedness OR pandemic response OR public health policy OR health policy OR public policy OR social policy OR global health policy OR public health decision-making OR regulations OR risk management OR risk prevention OR policy response OR pandemic control OR pandemic management OR disaster management OR disaster governance OR epidemic response OR epidemic management OR epidemic governance OR emergency response OR emergency management OR public health measures OR public health interventions ) AND SU covid |
|  |  |

Note- Filters and Boolean Search was applied at Title filter and all Fields.  Additionally, to force databases to search for an exact phrase, we used database specific measures for a more specific search with more precision, and not as disparate words. COVID and other Mesh terms were included in All Fields (Pubmed), All Text (EBSCO) and Keywords (Global Health) to ensure that the term COVID appeared in the publication.

**Supplementary Table 2. Inclusion and exclusion criteria for identifying studies (governance and decision-making scoping review, global, 2020-2022).**

| **Inclusion Criteria** | **Exclusion Criteria** |
| --- | --- |
| ● **Timeframe**: Publication date (online or print) between Jan 1, 2020 – Jan 31, 2022  ● **Language**: English  ● **Study design**: Published, peer reviewed primary/empirical data (qualitative and quantitative data) including from:  ✔ descriptive studies,  ✔ case studies and case series,  ✔ research articles (derives data from policy observations, interviews, or verbal interactions etc.)  ● **Outcome**: Description and/or analysis of governance and decision-making processes in the COVID-19 pandemic/health emergency at the global, national, subnational, and local level:  o Overarching pandemic/health emergency governance  o Decisions to detect/trace/break transmission of virus  o Decisions to ensure testing, treatment facilities, access to essential medical products for people  o Decisions to facilitate people’s adherence/acceptance to public health strategies (like travel restrictions, use of facemask, vaccine certificates etc.) and communicate with public  o Decisions to support the economy, trade, and community activities | ● Not empirical research, including:  × clinical guideline documents,  × news articles,  × reviews (systematic reviews, literature reviews, narrative reviews)  × commentaries,  × editorials,  × reports  × white papers,  × background papers,  × guideline documents (those from international organizations, bodies etc.)  ● Studies focused on clinical decision-making:  × Single hospital guidelines for treatment of other diseases (cancer, dental practices)  × Guidelines from clinical organizations like American Medical Association about changes in practices  ● Studies focused on policy implementation and changes to address the impact of the pandemic on other public health issues/clinical care issues (opioid epidemic, suspension of elective surgeries etc.)  ● Pre-print articles (such as those published in bioRxiv & medRxiv)  ● Full text not available  ● Abstract in English but the main body of the article not in English |

**Supplementary Table 3. Summary of publications (n=24) included in our review by country of focus, author affiliation, publication type, methodological approach, analysis and conceptualization of governance (governance and decision-making scoping review, global, 2020-2022).**

| **No.** | **Title** | **Geographic Region** | **Country** | **HIC/LMIC** | **Author** | | **Publication Type** | **Methodological Approach** | **Data Collection Methods** | **Level of Governance** | **Aspect of Governance** | **Governance conceptualization** |
| --- | --- | --- | --- | --- | --- | --- | --- | --- | --- | --- | --- | --- |
|  |  |  |  |  | **Country** | **Organization** |  |  |  |  |  |  |
| 1 | Assessing the South Korean Model of Emergency Management during the COVID-19 Pandemic. | Western Pacific Region | South Korea | High Income | South Korea, United States of America, China, New Zealand | Academia, Government | Commentary with empirical data | Qualitative | In-depth interviews | National | Process | Yes |
| 2 | Bio-Politics and Calculative Technologies in COVID-19 Governance: Reflections From England. | European Region | United Kingdom | High Income | United Kingdom | Academia | Research article | Qualitative | Document analysis | National | Process | Yes |
| 3 | Collaborative Leadership, Collective Action, and Community Governance against Public Health Crises under Uncertainty: A Case Study of the Quanjingwan Community in China. | Western Pacific Region | China | Upper Middle Income | China | Academia | Research article | Qualitative | Participant observations and unstructured interviews | Community | Process | Yes |
| 4 | Collaborative neighborhood governance and its effectiveness in community mitigation to COVID-19 pandemic: From the perspective of community workers in six Chinese cities. | Western Pacific Region | China | Upper Middle Income | China | Academia | Research article | Quantitative | Large-scale questionnaire survey | Sub-national | Determinant | Yes |
| 5 | Community Resilience Governance on Public Health Crisis in China. | Western Pacific Region | China | Upper Middle Income | China | Academia | Research article | Mixed | Questionnaire & interviews | Sub-national | Process | Yes |
| 6 | COVID-19 pandemic control and administrative issues in Pakistan: How Pakistan mitigated both pandemic and administration issues? | Eastern Mediterranean Region | Pakistan | Lower middle income | China | Academia | Research article | Mixed | Modeling | National | Process | Yes |
| 7 | COVID-19 Policy Response and the Rise of the Sub-National Governments. | Region of the Americas | Canada, United States of America | High Income | United States of America, Canada, United Kingdom | Academia, No Affiliation | Research article | Mixed | Statistical analysis | National & subnational | Process | No |
| 8 | Efficiency in the governance of the Covid-19 pandemic: political and territorial factors. | European Region | Multiple countries | Not applicable | Spain | Academia | Research article | Quantitative | Statistical analysis/regression | National | Performance | No |
| 9 | From Poor to Worse: Health Policy and Politics Scholars’ Assessment of the U.S. COVID‐19 Response and Its Implications. | Region of the Americas | United States of America | High Income | United States of America | Academia | Research article | Quantitative | Survey | National | Process | No |
| 10 | From response to transformation: how countries can strengthen national pandemic preparedness and response systems. | Multiple | Multiple countries | Not applicable | United Kingdom, Canada, United States of America, Singapore | Academia, Independent panel | Research article | Mixed | Interviews & submissions | National | Performance | No |
| 11 | Global health security must embrace a One Health approach: Contributions and experiences of veterinarians during the COVID-19 response in Australia. | Western Pacific Region. | Australia | High Income | Australia, United Kingdom | Academia | Research article | Qualitative | Interviews | National | Process | No |
| 12 | How regime type and governance quality affect policy responses to COVID-19: A preliminary analysis. | Multiple | Multiple countries | Not applicable | Thailand | Academia, Government | Research article | Quantitative | regression, correlation | National | Determinant | No |
| 13 | In the name of COVID-19: legitimizing the exclusion of community participation in Ecuador’s health policy: Special call: Health Promotion Perspectives on the COVID-19 Pandemic. | Region of the Americas | Ecuador | High Income | Ecuador, United States of America | CSO, Academia | Research article | Mixed | line by line coding and tabulation | National | Process | Yes |
| 14 | Italian mayors and the management of COVID-19: adaptive leadership for organizing local governance. | European Region | Italy | High Income | Italy, United Kingdom | Academia | Research article | Qualitative | Qualitative questionnaire survey | Sub-national | Determinant | Yes |
| 15 | ‘Let communities do their work’: the role of mutual aid and self-help groups in the Covid-19 pandemic response. | African Region, Eastern Mediterranean Region | Kenya, Sudan, Philippines | Lower middle income, Upper Middle Income | Denmark, United Kingdom | Academia, International Non-Governmental Organizations | Research article | Mixed | Document review, database analysis & interviews | National | Process | No |
| 16 | Moonshots or a cautious take-off? How the Big Five leadership traits predict Covid-19 policy response. | Multiple | Multiple countries | Not applicable | United Kingdom | Academia | Research article | Quantitative | pre-trained machine classifier | National | Determinant | Yes |
| 17 | Multi-level governance tackling the COVID-19 pandemic in China. | Western Pacific Region | China | Upper Middle Income | Portugal | Academia | Research article | Mixed | case study | National | Process | Yes |
| 18 | National health governance, science and the media: drivers of COVID-19 responses in Germany, Sweden and the UK in 2020. | European Region | Germany, Sweden, United Kingdom | High Income | Sweden, United Kingdom, Germany | Academia | Research article | Qualitative | systematic document and policy analysis | National | Determinant | Yes |
| 19 | Pandemic preparedness systems and diverging COVID-19 responses within similar public health regimes: a comparative study of expert perceptions of pandemic response in Denmark, Norway, and Sweden. | European Region | Denmark, Norway, Sweden | High Income | Denmark | Academia | Research article | Mixed | case comparison, expert perception survey | National | Process | No |
| 20 | Pandemic response in rural Peru: Multi-scale institutional analysis of the COVID-19 crisis. | Region of the Americas | Peru | Upper Middle Income | Peru, United States of America | Academia | Research article | Qualitative | interviews | National, regional, subnational, local | Process | Yes |
| 21 | Responses to COVID-19: The role of governance, healthcare infrastructure, and learning from past pandemics. | Multiple | Multiple countries | Not applicable | United States of America, India | Academia | Research article | Quantitative | bivariate correlation and descriptive statistics | National | Determinant | Yes |
| 22 | Six Cs of pandemic emergency management: A case study of Taiwan’s initial response to the COVID-19 pandemic. | Not applicable | Taiwan | Not applicable | United States of America, Taiwan | Academia | Research article/Case Study | Mixed | case analysis, and online public survey | National | Determinant | Yes |
| 23 | The local governance of COVID-19: Disease prevention and social security in rural India. | South-East Asia Region | India | Lower middle income | Norway, Sweden | Academia | Research article | Qualitative | Interviews and policy documents review | Community | Process | Yes |
| 24 | The role of good governance in the race for global vaccination during the COVID-19 pandemic. | Multiple | Multiple countries | Not applicable | United States of America, Iran | Academia | Research article | Quantitative | Statistical analysis | National | Determinant | Yes |
